# Diet–Microbiome Relationships in Prostate-Cancer Survivors with Prior Androgen Deprivation-Therapy Exposure and Previous Exercise Intervention Enrollment

**DOI:** 10.3390/microorganisms14010251

**Published:** 2026-01-21

**Authors:** Jacob Raber, Abigail O’Niel, Kristin D. Kasschau, Alexandra Pederson, Naomi Robinson, Carolyn Guidarelli, Christopher Chalmers, Kerri Winters-Stone, Thomas J. Sharpton

**Affiliations:** 1 Department of Behavioral Neuroscience, Oregon Health & Science University, Portland, OR 97239, USA; oniela@ohsu.edu (A.O.); alexcpederson@gmail.com (A.P.); robinnao@ohsu.edu (N.R.); 2 Departments of Neurology and Radiation Medicine, Division of Neuroscience, ONPRC, Oregon Health & Science University, Portland, OR 97239, USA; 3 Knight Cancer Institute, Oregon Health and Science University, Portland, OR 97239, USA; borsch@ohsu.edu (C.G.); chalmech@ohsu.edu (C.C.); wintersk@ohsu.edu (K.W.-S.); 4 Department of Microbiology, Oregon State University, Corvallis, OR 97331, USA; kristin.kasschau@oregonstate.edu; 5 Division of Oncological Sciences, School of Medicine, Oregon Health and Science University, Portland, OR 97239, USA; 6 Department of Statistics, Oregon State University, Corvallis, OR 97331, USA; 7 Linus Pauling Institute, Oregon State University, Corvallis, OR 97331, USA

**Keywords:** diet, exercise intervention, androgen suppression, cognition, prostate-cancer survivorship, MEDAS, HEI-2015, MIND, *APOE* genotype

## Abstract

The gut microbiome is a modifiable factor in cancer survivorship. Diet represents the most practical intervention for modulating the gut microbiome. However, diet–microbiome relationships in prostate-cancer survivors remain poorly characterized. We conducted a comprehensive analysis of diet–microbiome associations in 79 prostate-cancer survivors (ages 62–81) enrolled in a randomized exercise intervention trial, 59.5% of whom still have active metastatic disease. Dietary intake was assessed using the Diet History Questionnaire (201 variables) and analyzed using three validated dietary pattern scores: Mediterranean Diet Adherence Score (MEDAS), Healthy Eating Index-2015 (HEI-2015), and the Mediterranean-Dash Intervention for Neurodegenerative Delay (MIND) diet score. Gut microbiome composition was characterized via 16S rRNA sequencing. Dimensionality reduction strategies, including theory-driven diet scores and data-driven machine learning (Random Forest, and Least Absolute Shrinkage and Selection Operator (LASSO)), were used. Statistical analyses included beta regression for alpha diversity, Permutational Multivariate Analysis of Variance (PERMANOVA) for beta diversity (both Bray–Curtis and Sørensen metrics), and Microbiome Multivariable Associations with Linear Models (MaAsLin2) with negative binomial regression for taxa-level associations. All models tested interactions with exercise intervention, *APOLIPOPROTEIN E* (*APOE*) genotype, and testosterone levels. There was an interaction between MEDAS and exercise type on gut alpha diversity (Shannon: *p* = 0.0022), with stronger diet–diversity associations in strength training and Tai Chi groups than flexibility controls. All three diet-quality scores predicted beta diversity (HEI *p* = 0.002; MIND *p* = 0.025; MEDAS *p* = 0.034) but not Bray–Curtis (abundance-weighted) distance, suggesting diet shapes community membership rather than relative abundances. Taxa-level analysis revealed 129 genera with diet associations or diet × host factor interactions. Among 297 dietary variables tested for cognitive outcomes, only caffeine significantly predicted Montreal Cognitive Assessment (MoCA) scores after False Discovery Rate (FDR) correction (*p* = 0.0009, *q* = 0.014) through direct pathways beneficial to cognitive performance without notable gut microbiome modulation. In cancer survivors, dietary recommendations should be tailored to exercise habits, genetic background, and hormonal status.

## 1. Introduction

Cancer survivors often experience behavioral and cognitive changes [1,2]. Prostate cancer is highly survivable, but patients suffer from the effects of the cancer and cancer treatment [3]. Apolipoprotein E (apoE) is involved in cholesterol and lipid homeostasis [4], immunomodulation [5], and apoE expression is increased in prostate cancer and correlate with poor prognosis [6]. In humans, there are three major isoforms of apoE, E2, E3, and E4. E4 is associated with increased susceptibility to develop cognitive injury after cancer treatment in breast-cancer, lymphoma, and testicular-cancer patients [7].

Androgen-deprivation therapy (ADT), often used in prostate-cancer patients, can increase cardiovascular risk [8], diabetes risk, osteoporosis risk [9], cognitive injury [10,11], and risk to develop Alzheimer’s disease (AD) [12]. Exercise intervention might improve quality of life in survivors [13,14] but might increase salivary testosterone levels. In addition, the gut microbiome can generate testosterone and, as a result, promote prostate-cancer growth and resistance in prostate-cancer patients who received ADT [15,16].

The gut microbiome interacts with the brain [17] via the gut–brain axis, and apoE might be involved in the regulation of this axis in cancer patients, as it is expressed in the gut [18]; modulates inflammation and gastrointestinal health [19]; and is linked to antitumor activity [20,21], survival in melanoma patients, and risk to develop gastric cancers [20]. The beneficial effects of exercise intervention on the brain might involve alterations in the gut microbiome and gut–brain axis [22,23], and these effects might be *APOE* genotype-dependent. We recently reported that exercise intervention, *APOE* genotype, and salivary testosterone levels modulate gut microbiome–cognition association in prostate-cancer survivors [24].

In addition to exercise, diet has a strong effect on the gut microbiome [25,26,27,28,29]. The Diet Questionnaire III (DHQ) from the National Cancer Institute for those over 19 years of age and based on 24 h dietary recall data from the National Health and Nutrition Examination Surveys [30] is often used to assess diets in study participants. However, as it contains 201 variables, its use in integrated diet–gut microbiome analyses with limited sample sizes is statistically challenging. Therefore, diet indices/scores have been proposed [31,32], especially considering those related to the Mediterranean, diet which has consistently been shown to be beneficial for gut [33] and brain health [34]. In a recent systematic review, the Mediterranean diet was not consistently linked to reduced prostate-cancer risk [35]. This might be due to modulating effects of genetic factors.

In this study, we conducted a comprehensive analysis of diet–microbiome associations in prostate-cancer survivors enrolled in a randomized exercise intervention trial using diet scores associated with improved cognitive performance: the Mediterranean Diet Adherence Score (MEDAS) [34,36], Healthy Eating Index-2015 (HEI-2015) [37,38], and the Mediterranean-Dash Intervention for Neurodegenerative Delay (MIND) [34,39,40,41] diet scores.

## 2. Materials and Methods

### 2.1. Subject Recruitment, Study Design, and Study Participants

This study represents a secondary analysis of dietary intake and gut microbiome composition in prostate-cancer survivors enrolled in a randomized exercise intervention trial [24,42]. The parent studies evaluated the effects of exercise modality on fall risk, cognitive function, and physiological outcomes in men with a history of androgen-deprivation therapy (ADT) for prostate cancer. The present analysis examines associations between habitual dietary intake and gut microbiome diversity and composition, with attention to effect modification by exercise intervention, *APOE* genotype, and testosterone status. Men were eligible if they had a confirmed diagnosis of prostate cancer, had received ADT (currently or within the past 10 years), were aged 55 years or older, were able to provide informed consent, and had no contraindications to moderate-intensity exercise. Exclusion criteria included unstable cardiovascular disease, neurological conditions affecting cognition or balance, current chemotherapy, and antibiotic use within 30 days of stool sample collection. A total of 79 prostate-cancer survivors with complete microbiome data were included. The cohort was predominantly elderly (age range: 62–81 years), with 59.5% (*n* = 47) having metastatic disease. All participants had ADT exposure, creating a relatively homogeneous hormonal milieu. Participants were randomized to one of three 12-week exercise interventions: progressive resistance training (*n* = 36, 45.6%), Tai Chi (*n* = 23, 29.1%), or flexibility and stretching exercises (control; *n* = 20, 25.3%). Dietary assessment and biological sample collection occurred at baseline, prior to initiation of the exercise intervention. Exercise group assignment served as a potential effect modifier of diet–microbiome associations. This study was approved by the Oregon Health and Science University Institutional Review Board, and all participants provided written informed consent.

### 2.2. Cognitive Testing

The Montreal Cognitive Assessment [43] was administered through videoconferencing, as reported in [24].

### 2.3. Dietary Assessment

Habitual dietary intake was assessed using the National Cancer Institute Diet History Questionnaire III (DHQ-III), a validated food frequency questionnaire capturing usual dietary intake over the preceding 12 months [30]. The DHQ-III yields estimates for 201 dietary variables, including macronutrients (energy, protein, carbohydrates, total fat, saturated fat, monounsaturated fatty acids, and polyunsaturated fatty acids); micronutrients (vitamins A, C, D, E, K, and B-complex; and minerals, including calcium, iron, magnesium, zinc, and selenium); food groups (fruits, vegetables, whole grains, dairy, and protein foods); and bioactive compounds (dietary fiber, carotenoids, flavonoids, and caffeine). Missing values were handled via complete-case analysis for each model.

Given the high dimensionality of dietary data (201 variables) relative to sample size (*n* = 79), we employed both theory-driven dietary-pattern scores and data-driven feature selection. The 13-item Mediterranean Diet Adherence Screener (MEDAS) was calculated according to Schroder et al. [44], with one point assigned for each dietary criterion met (total range, 0–13). The Healthy Eating Index-2015 (HEI-2015) was calculated using the density-based scoring method [45], comprising nine adequacy components and four moderation components (total range, 0–100). The Mediterranean-DASH Intervention for Neurodegenerative Delay (MIND) diet score was calculated according to Morris et al. [46], comprising 10 brain-healthy and 5 brain-unhealthy food groups scored based on consumption frequency (total range, 0–15).

Data-driven feature selection employed Random Forest models fit using all 201 dietary variables as predictors of Shannon diversity, with variable importance assessed using permutation-based scores. Cross-validated LASSO regression was performed using the glmnet package [47] with 10-fold cross-validation, selecting the optimal regularization parameter to minimize cross-validated mean squared error.

### 2.4. Sample Collection of Saliva and Stool Samples

Stool was collected for gut microbiome analysis, and saliva samples were collected for *APOE* genotyping and analysis of salivary testosterone (T) levels, as reported in [24]. Briefly, participants collected stool samples at home using OMNIgene-GUT tubes (DNA Genotek, Ottawa, ON, Canada) for DNA stabilization, returning samples via mail at ambient temperature. Samples were stored at −80 °C upon receipt. Saliva samples were collected using Oragene-DNA kits (DNA Genotek) for *APOE* genotyping and testosterone measurement. Microbial community DNA was extracted using QIAamp PowerFecal Pro DNA kits (Qiagen, Hilden, Germany) according to manufacturer instructions, with concentration and purity assessed using a NanoDrop spectrophotometer (Thermo Fisher Scientific, Waltham, MA, USA).

The V4 hypervariable region of the 16S rRNA gene was amplified using the 515F/806Rb primer pair with Illumina adapter sequences. PCR amplification was performed in triplicate, with replicates pooled prior to sequencing. Amplicon libraries were sequenced on an Illumina MiSeq platform (v3 chemistry; 2 × 300 bp paired-end reads) at the Center for Quantitative Life Sciences, Oregon State University, targeting a minimum of 40,000 reads per sample. Quality control included ATCC 20-strain staggered mix mock communities to assess taxonomic assignment accuracy and molecular-grade water blanks to monitor contamination.

Salivary testosterone levels were assessed using Salimetrix ELISAs, State College, PA, USA.

### 2.5. Bioinformatic Processing

Demultiplexed sequences were processed using the DADA2 pipeline [48] in R (version 4.2). Processing included primer trimming using cutadapt [49], quality filtering and trimming, error rate learning and denoising, paired-end read merging, chimera removal using the consensus method, and amplicon sequence variant (ASV) inference. Taxonomy was assigned using the naive Bayesian classifier in DADA2 against the SILVA database (version 138) [50]. A phylogenetic tree was constructed using FastTree [51]. Microbiome data were organized into a phyloseq object [52] containing the ASV abundance table, sample metadata, taxonomic assignments, and phylogenetic tree.

### 2.6. Host Factor Assessment

*APOE* genotype was determined from saliva DNA using TaqMan SNP genotyping assays (Thermo Fisher Scientific) targeting rs429358 and rs7412 polymorphisms (rs429358 (C_3084793_20), which distinguish the ε4 (C) from ε2/ε3; rs7412 (C_904973_10) distinguishes the ε2 (T) from ε3/ε4 (see also Supplementary Materials Table S1)). Genotypes were classified as E2 carriers (e2/e2 or e2/e3; *n* = 7), E3/E3 homozygotes (*n* = 46), or E4 carriers (e3/e4 or e4/e4; *n* = 17); nine participants had missing genotype data. Salivary testosterone was measured using enzyme-linked immunosorbent assay (ELISA) and log-transformed prior to analysis. Cognitive function was assessed using the Montreal Cognitive Assessment (MoCA) [43], administered via secure videoconferencing by trained research staff. Total MoCA scores range from 0 to 30, with scores below 26 generally indicating cognitive impairment.

### 2.7. Statistical Analysis

All analyses were performed in R (version 4.2) with a fixed random seed. Statistical significance was defined as *p* < 0.05, with false discovery rate (FDR) correction applied for multiple comparisons. Alpha diversity was calculated using Shannon diversity index, Simpson diversity index, and observed ASV richness on rarefied count data using the phyloseq package. Associations between dietary exposures and alpha diversity were evaluated using beta regression (betareg package [53]) for bounded indices and linear regression for richness. Models included diet score, exercise group, *APOE* status, age, and log-transformed testosterone as covariates. Interaction terms (diet score x exercise; diet score x *APOE*) were tested separately.

Beta diversity was assessed using Bray–Curtis dissimilarity (abundance-weighted) and Sørensen distance (presence/absence) computed using the vegan package [54]. Associations between dietary exposures and community composition were tested using PERMANOVA via the adonis2 function [55] with 999 permutations. Constrained ordination was performed using distance-based redundancy analysis (db-RDA) via the capscale function.

Taxon-level associations were tested using MaAsLin2 [56] with negative binomial regression on raw counts. Taxa present in fewer than 10% of samples or with mean relative abundance below 0.01% were excluded. Multiple testing correction used the Benjamini–Hochberg FDR method [57], with *q* < 0.10 considered significant and *q* < 0.25 reported as suggestive.

Diet–cognition associations were evaluated by testing all 297 dietary variables (201 DHQ-III variables, 3 diet-pattern scores, 41 component scores, and 52 derived variables) against MoCA scores using linear regression adjusted for exercise group, *APOE* status, age, and testosterone. Variables surviving FDR correction were further evaluated in the E3/E3 subgroup. Mediation analysis testing whether diet–cognition associations were mediated through gut microbiome diversity was conducted using the mediation package [58], estimating average causal mediation effect (ACME), average direct effect (ADE), and proportion mediated using 1000 bootstrap simulations.

Key R packages included phyloseq (v1.40+) for microbiome data structures, vegan (v2.6+) for diversity metrics and PERMANOVA, MaAsLin2 (v1.10+) for taxon-level associations, betareg (v3.1+) for beta regression, glmnet (v4.1+) for LASSO regression, randomForest (v4.7+) for feature selection, and mediation (v4.5+) for mediation analysis.

## 3. Results

### 3.1. Cohort Characteristics and Baseline Assessments

This analysis included 79 prostate-cancer survivors with complete microbiome and dietary data. The cohort was elderly (age range: 62–81 years) and predominantly affected by metastatic disease (59.5%, *n* = 47). All participants had prior exposure to androgen-deprivation therapy (ADT), creating a relatively homogeneous hormonal milieu. Participants were randomized across three exercise interventions: strength training (*n* = 36, 45.6%), Tai Chi (*n* = 23, 29.1%), and flexibility control (*n* = 20, 25.3%). *APOE* genotype was available for 70 participants (88.6%), distributed as E3/E3 homozygotes (*n* = 46, 65.7%), E4 carriers (*n* = 17, 24.3%), and E2 carriers (*n* = 7, 10.0%). Testosterone measurements were available for 73 participants (92.4%; log-transformed mean ± SD: 3.83 ± 0.87).

**Figure 1:**
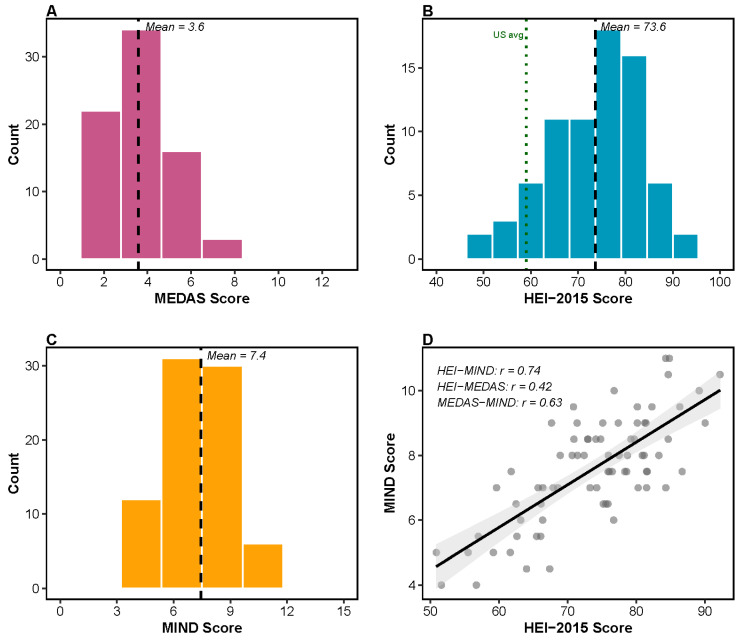
Diet score distributions in prostate-cancer survivors. Distribution of diet-quality scores among study participants (*n* = 79). (**A**) Mediterranean Diet Adherence Score (MEDAS; possible range, 0–13); mean = 3.6, indicating relatively low Mediterranean diet adherence, with the cohort utilizing 54% of the possible score range. (**B**) Healthy Eating Index-2015 (HEI-2015; possible range, 0–100); mean = 73.6, exceeding the US national average (~59, green dotted line). (**C**) MIND diet score (possible range, 0–15); mean = 7.4, consistent with published values in older adult populations. (**D**) Correlation scatterplot showing positive associations among diet scores (HEI-MIND, *r* = 0.74; HEI-MEDAS, *r* = 0.42; and MEDAS-MIND, *r* = 0.63), indicating convergent validity while measuring distinct dietary aspects. Dashed lines indicate cohort means.

Dietary pattern scores showed moderate variation across the cohort (Figure 1). Mediterranean Diet Adherence Score (MEDAS) ranged from 1 to 8 (mean ± SD: 3.59 ± 1.55; *n* = 75), indicating relatively low Mediterranean diet adherence, with the cohort utilizing approximately half of the possible 0–13 score range (Figure 1A). In contrast, Healthy Eating Index-2015 (HEI-2015) scores ranged from 50.9 to 92.2 (mean ± SD: 73.64 ± 9.22; *n* = 75), indicating generally good overall diet quality, exceeding the US national average of approximately 59 points (Figure 1B). MIND diet scores ranged from 4 to 11 (mean ± SD: 7.44 ± 1.69; *n* = 79), consistent with published values in older adult populations (Figure 1C). The three diet scores were moderately to strongly correlated with one another (HEI-2015 and MIND, *r* = 0.74; MEDAS and MIND, *r* = 0.63; and HEI-2015 and MEDAS, *r* = 0.42) (Figure 1D), suggesting they capture overlapping but distinct aspects of diet quality.

Among individual dietary variables, caffeine intake exhibited the highest coefficient of variation (CV = 79.4%; mean ± SD, 249 ± 198 mg/day; and range, 0.2–962 mg/day), whereas diet-pattern scores showed more restricted variation (CV: 12.5–43.3%).

**Figure 2:**
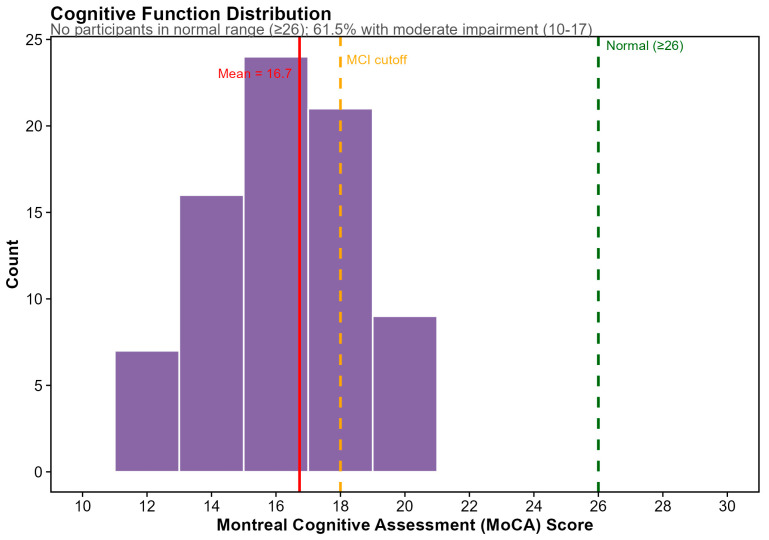
Study cohort distribution of Montreal Cognitive Assessment (MoCA) scores among study participants (*n* = 78 with available data). Scores ranged from 11 to 21 (mean = 16.7), representing only one-third of the possible score range (0–30). Green dashed line indicates normal cognition cutoff (≥26); no participants scored in the normal range. Orange dashed line indicates mild cognitive impairment (MCI) cutoff (<18); 61.5% of participants (*n* = 48) scored in the moderate impairment range (10–17), while 38.5% (*n* = 30) met criteria for MCI (18–25). This restricted variance reflects the cognitive effects of androgen-deprivation therapy in this cancer-survivor population.

Cognitive function, assessed via Montreal Cognitive Assessment (MoCA), was available for 78 participants (98.7%). Scores ranged from 11 to 21 (mean ± SD: 16.73 ± 2.34), representing only one-third of the possible 0–30 score range. Notably, no participants scored in the normal cognitive range (≥26), with 38.5% (*n* = 30) meeting criteria for mild cognitive impairment (MoCA 18–25), and 61.5% (*n* = 48) meeting criteria for moderate impairment (MoCA 10–17) (Figure 2). The observed restricted variance and universal cognitive impairment likely reflect the effects of ADT on cognition in this population, a constraint that informed our subsequent analyses of diet–cognition relationships.

### 3.2. Diet–Microbiome Associations: Alpha Diversity

**Figure 3:**
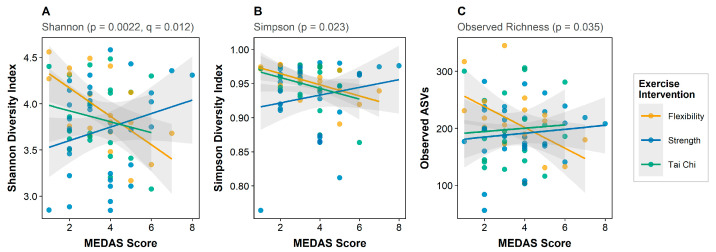
Mediterranean diet × exercise interaction on gut microbiome alpha diversity. Association between Mediterranean Diet Adherence Score (MEDAS) and alpha diversity metrics stratified by exercise intervention group (*n* = 75 with complete data). (**A**) Shannon diversity index (interaction *p* = 0.0022; FDR *q* = 0.012). (**B**) Simpson diversity index (interaction *p* = 0.023). (**C**) Observed ASV richness (interaction *p* = 0.035). The significant interactions indicate that Mediterranean diet adherence shows stronger positive associations with microbiome diversity in physically active groups (strength training, blue; Tai Chi, green) compared to the sedentary control group (flexibility, orange). The consistent interaction pattern across all three alpha diversity metrics provides robust evidence that Mediterranean diet effects on gut microbiome diversity depend on physical-activity status. Points represent individual participants; lines represent linear regression fits with 95% confidence intervals.

A striking interaction between Mediterranean diet adherence and exercise intervention emerged for alpha diversity. This interaction was significant across all metrics, with the strongest effect observed for Shannon diversity (interaction β = +0.55, SE = 0.18, *p* = 0.0022, FDR *q* = 0.012) (Figure 3), and consistent effects for Simpson diversity (*p* = 0.023) and observed richness (*p* = 0.035) (Figure 3). The interaction pattern indicates that Mediterranean diet adherence shows stronger positive associations with microbiome diversity in the strength training and Tai Chi groups compared to the flexibility control group. This finding suggests that physical activity may enhance the gut microbiome’s responsiveness to dietary inputs, a pattern with potential implications for integrated lifestyle interventions in cancer survivorship.

### 3.3. Diet–Microbiome Associations: Beta Diversity

**Figure 4:**
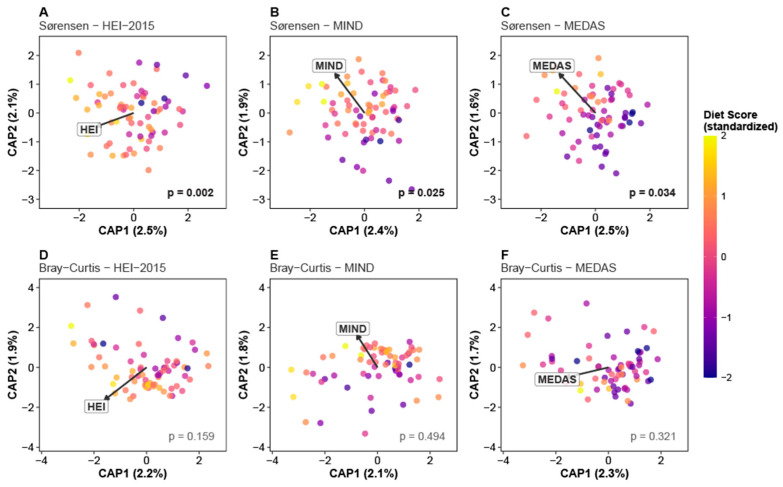
Constrained ordinations reveal metric-specific diet–beta diversity associations. Canonical Analysis of Principal Coordinates (CAP; also termed distance-based redundancy analysis, db-RDA) comparing diet score associations with presence/absence (Sørensen) versus abundance-weighted (Bray–Curtis) beta diversity. (**A**–**C**) Sørensen distance ordinations showing significant associations for all three diet scores ((**A**) HEI-2015 *p* = 0.002; (**B**) MIND *p* = 0.025; and (**C**) MEDAS *p* = 0.034). (**D**–**F**) Bray–Curtis distance ordinations showing non-significant associations for all diet scores ((**D**) HEI-2015 *p* = 0.159; (**E**) MIND *p* = 0.494; and (**F**) MEDAS *p* = 0.321). Points represent individual participants (*n* = 69 with complete data), colored by standardized diet score (plasma color scale). The biplot vector in each panel indicates the direction and relative magnitude of the diet score loading in ordination space. PERMANOVA *p*-values are displayed in the lower right of each panel (significant values in bold black; non-significant in gray). Axis labels show percent of total variance explained by each constrained axis. All models adjusted for exercise intervention and APOE genotype. This dissociation between distance metrics suggests that diet quality shapes community membership (which taxa are present) rather than relative abundance patterns (how much of each taxon), consistent with a model where plant-rich diets provide diverse substrates that enable specialist taxa to colonize without preferentially fueling blooms of particular organisms.

Analysis of between-sample (beta) diversity revealed a striking dissociation between the two metrics examined (Figure 4). All three diet-quality scores significantly predicted Sørensen (presence/absence) distance—HEI-2015 (*R*^2^ = 2.3%, *p* = 0.002), MIND (*R*^2^ = 2.0%, *p* = 0.025), and MEDAS (*R*^2^ = 1.9%, *p* = 0.034)—effects that remained significant after adjustment for exercise group and *APOE* status. In marked contrast, none of the diet-quality scores significantly predicted Bray–Curtis (abundance-weighted) distance: HEI-2015 (*R*^2^ = 1.7%, *p* = 0.159), MIND (*R*^2^ = 1.4%, *p* = 0.494), and MEDAS (*R*^2^ = 1.5%, *p* = 0.321) (Figure 4). This metric-specific dissociation suggests that diet quality primarily affects community membership—which taxa are present—rather than relative abundance patterns among established taxa.

Random Forest feature selection identified specific dietary variables associated with beta diversity beyond the aggregate diet scores. For Bray–Curtis distance, meat/poultry/eggs intake showed the strongest association (*R*^2^ = 3.7%, *F* = 2.80, *p* = 0.004, FDR *q* < 0.05). For Sørensen distance, fruit intake was significantly associated with community membership (*R*^2^ = 2.3%, *F* = 1.70, *p* = 0.029, FDR *q* < 0.10). The MIND diet score showed a significant interaction with exercise intervention on Sørensen distance (*p* = 0.030), with HEI-2015 showing a trend toward interaction (*p* = 0.076), while no significant diet × exercise interactions were observed for Bray–Curtis distance (all *p* > 0.67).

### 3.4. Taxon-Level Associations

**Figure 5:**
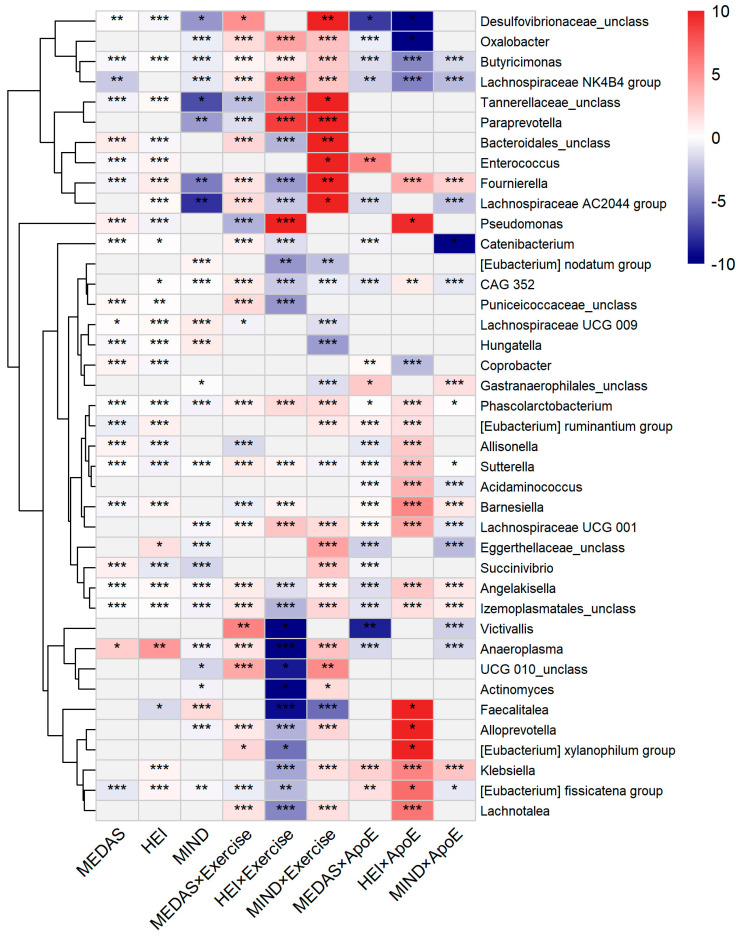
Consolidated diet–taxa associations and interactions (129 unique genera). Columns: Main effects (MEDAS, 41; HEI, 49; and MIND, 39 significant genera) followed by diet × exercise interactions (6 columns) and diet × ApoE interactions (6 columns). Color = coefficient from MaAsLin2 NEGBIN models (red = positive; blue = negative). Gray cells = not significant (*q* ≥ 0.1). Asterisks: * *q* < 0.1, ** *q* < 0.01, *** *q* < 0.001.

MaAsLin2 analysis using negative binomial regression identified extensive associations between diet-quality scores and genus-level abundances (Figure 5). At FDR *q* < 0.10, significant associations emerged for MEDAS (41 genera), HEI-2015 (49 genera), and MIND (39 genera). The most responsive genera included known health-associated taxa such as *Phascolarctobacterium* (a short-chain fatty acid producer), members of the Lachnospiraceae family, and *Butyricimonas*. Both positive and negative associations were observed, indicating that diet-quality scores promote certain taxa while suppressing others.

These taxon-level effects were substantially modified by host factors. Diet × exercise interactions (FDR *q* < 0.10) affected 55 genera for MEDAS, 48 genera for HEI, and 42 genera for MIND. For MEDAS × exercise interactions specifically, top responding genera included *Prevotella_9* (strongest MEDAS × Tai Chi interaction), *Phascolarctobacterium* (consistent MEDAS × strength training effects), and *Anaeroplasma* (strong exercise-dependent responses). Diet effects were also modified by *APOE* genotype (40–41 genera per diet score, *q* < 0.10) and testosterone status (34–51 genera per diet score), providing evidence for gene–diet–microbiome and hormone–diet–microbiome interactions in this cohort.

Across all main effect and interaction analyses, 129 unique genera showed at least one significant association (*q* < 0.10) with diet-quality scores or diet × host factor interactions. Several genera showed remarkable consistency, with *Angelakisella*, *Butyricimonas*, *Izemoplasmatales* (unclassified), *Phascolarctobacterium*, and *Sutterella* each showing significant effects across all nine analysis categories (three main effects plus six interaction categories). Given caffeine’s strong association with cognitive function (detailed below), we specifically tested for caffeine–taxa associations across 227 genera. After FDR correction, no significant associations emerged (lowest *q* = 0.69), indicating definitively null results for direct caffeine effects on gut microbial composition.

### 3.5. Dietary Predictors of Cognitive Function

**Figure 6:**
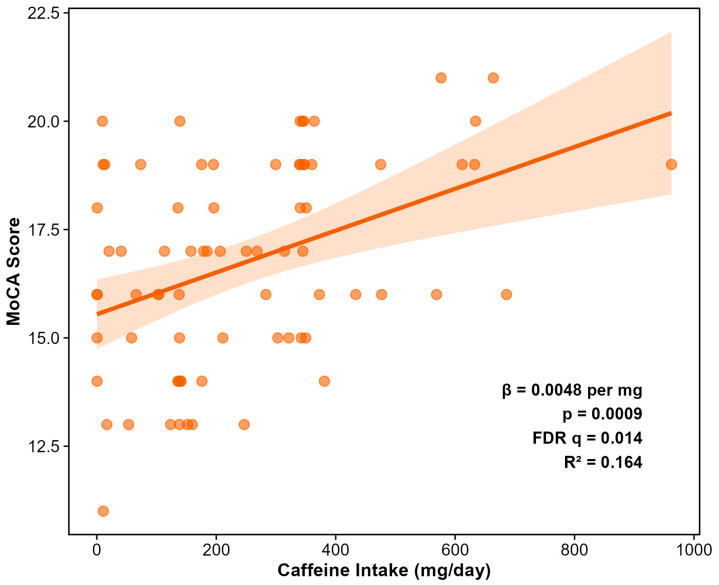
Positive association between daily caffeine intake and Montreal Cognitive Assessment (MoCA) score (*n* = 73 with complete data). Caffeine was the only dietary variable (of 297 tested) to achieve significance after FDR correction (β = +0.0047 per mg; *p* = 0.0009; FDR *q* = 0.014). The effect size corresponds to approximately 0.5 MoCA points per 100 mg daily caffeine (~1 cup of coffee). Points represent individual participants; line represents linear regression fit with 95% confidence interval.

**Figure 7:**
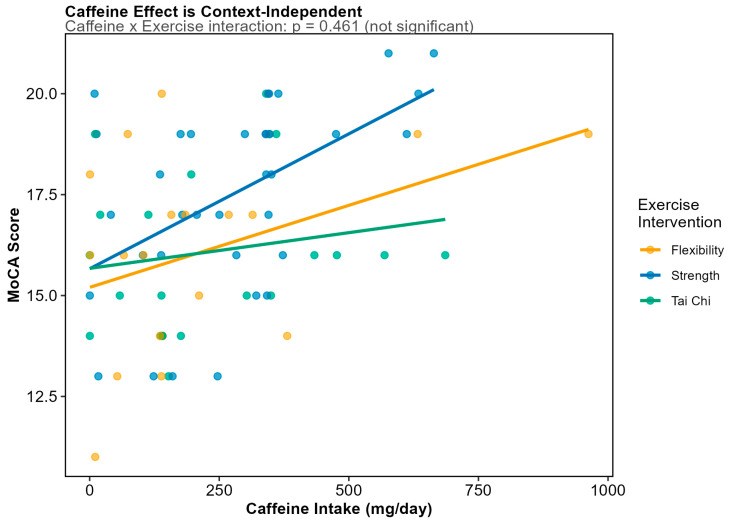
Association between caffeine intake and MoCA score stratified by exercise intervention group. Unlike the MEDAS × exercise interaction on microbiome diversity, the caffeine–cognition association does not show significant effect modification by exercise (interaction *p* = 0.461). This context-independent effect suggests that caffeine’s cognitive benefits operate through direct pharmacological mechanisms rather than through microbiome-mediated pathways that might be sensitive to exercise status.

Comprehensive feature selection across 297 dietary variables identified caffeine as the sole predictor of MoCA scores surviving FDR correction (β = +0.0047, SE = 0.0014, *p* = 0.0009, FDR *q* = 0.014) (Figure 6). This positive association indicates that higher caffeine intake is associated with better cognitive performance, with an effect size corresponding to approximately 0.5 MoCA points per 100 mg daily caffeine (roughly one cup of coffee). Both Random Forest and LASSO feature selection independently identified caffeine as the top dietary predictor of MoCA, with caffeine being the only dietary variable retained at the optimal regularization parameter (λ.1se) in cross-validated LASSO regression. The beneficial effect of caffeine was context-independent; there was no caffeine intake x exercise intervention (Figure 7).

Notably, none of the three diet-quality scores significantly predicted MoCA scores: MIND (*p* = 0.636), MEDAS (*p* = 0.652), or HEI-2015 (*p* = 0.622). This null finding is particularly striking for the MIND diet, which was explicitly designed for cognitive outcomes. However, the restricted MoCA variance in this cohort (observed range, 11–21 vs. possible 0–30; CV = 14%) and the universal cognitive impairment (0% with normal cognition) create a fundamentally different population context than the community-dwelling elderly cohorts in which the MIND diet was originally validated. The caffeine–MoCA association remained highly significant in the E3/E3 homozygous subgroup (*n* = 45, *p* = 0.0007), demonstrating robustness to genetic stratification and ruling out confounding by *APOE*-related differences in caffeine metabolism or cognition.

**Figure 8:**
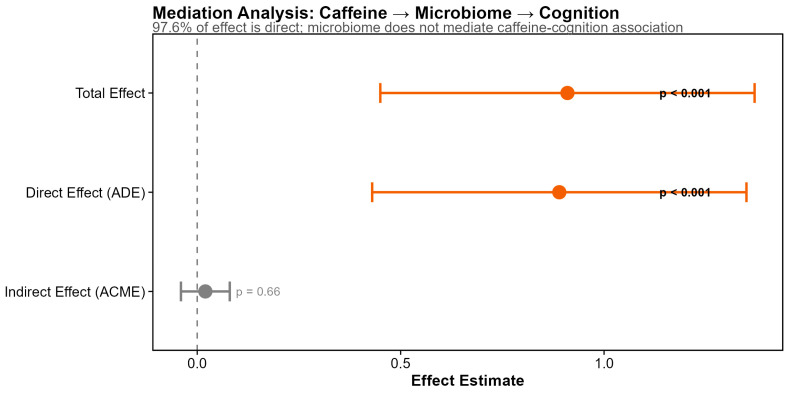
Mediation analysis: Caffeine effects on cognition are direct results of formal mediation analysis testing whether the caffeine–cognition association is mediated through gut microbiome alpha diversity (1000 bootstrap simulations). The average direct effect (ADE = 0.89; *p* < 0.001) was highly significant, while the average causal mediation effect (ACME = 0.02; *p* = 0.66) was not significant. The proportion mediated (2.4%) indicates that 97.6% of caffeine’s association with cognition operates through direct pathways, with negligible mediation through gut microbiome diversity.

Formal mediation analysis tested whether the caffeine–cognition association was mediated through gut microbiome alpha diversity (Figure 8). Using 1000 bootstrap simulations with standardized coefficients, we found that the total effect of caffeine on cognition (standardized β = 0.91, *p* < 0.001) was almost entirely attributable to the direct pathway (average direct effect = 0.89, *p* < 0.001), with negligible mediation through gut microbiome diversity (average causal mediation effect = 0.02, *p* = 0.66; proportion mediated = 2.4%, not significant) (Figure 6). Secondary mediation analyses testing specific taxa also failed to identify significant mediation pathways, though one genus showed a trend (indirect effect, *p* = 0.082). These results indicate that caffeine’s cognitive benefits in this cohort operate through direct neurological mechanisms rather than through modulation of the gut–brain axis.

## 4. Discussion

This study examined diet–microbiome associations in prostate-cancer survivors with prior ADT exposure, with attention paid to effect modification by exercise, *APOE* genotype, and testosterone status. Three principal findings emerged (see also the graphical abstract). First, diet–microbiome associations are context-dependent: the significant MEDAS × exercise interaction on alpha diversity (Shannon *p* = 0.0022) demonstrates that Mediterranean diet adherence has stronger positive associations with gut microbiome diversity in physically active individuals, supporting precision nutrition frameworks wherein dietary recommendations account for concurrent lifestyle factors. Second, diet quality shapes community membership rather than abundance dynamics: all three diet scores predicted Sørensen (presence/absence) but not Bray–Curtis (abundance-weighted) beta diversity, suggesting diet determines which taxa colonize rather than their relative abundances once established. Third, caffeine uniquely predicts cognitive function through direct pathways: among 297 dietary variables, only caffeine significantly predicted MoCA scores (*p* = 0.0009, *q* = 0.014), with mediation analysis confirming 97.6% of this effect operates independently of microbiome modulation.

The MEDAS x exercise interaction suggests that exercise may prime the gut ecosystem to respond to dietary inputs, consistent with the literature demonstrating that physical activity modulates gut microbiome composition through altered splanchnic blood flow, gut transit time, and systemic inflammation [22,23,59,60,61,62,63]. The specificity of this interaction (significant for MEDAS but not HEI or MIND) may reflect the Mediterranean diet’s characteristic substrates (monounsaturated fatty acids, fermentable fibers, and polyphenols) being particularly responsive to exercise-induced physiological changes, whereas HEI and MIND capture broader diet-quality constructs less mechanistically coupled to exercise pathways. This finding has direct clinical implications: dietary counseling for cancer survivors may be most effective when paired with exercise recommendations. A sedentary prostate-cancer survivor may derive less microbiome benefit from improving Mediterranean diet adherence than one simultaneously engaging in resistance training or Tai Chi, suggesting that oncology dietitians and exercise physiologists should coordinate rather than deliver recommendations in isolation. Such integrated counseling aligns with emerging precision nutrition frameworks [64].

The consistent pattern wherein all three diet scores predicted Sørensen but none predicted Bray–Curtis distance suggests a two-stage model: diet quality may primarily affect community membership by creating diverse ecological niches through varied fibers and polyphenols [65], enabling specialist taxa to colonize, while relative abundances of established taxa may be determined by other factors, including host genetics, immune function, or acute dietary variation. The finding that specific foods (meat/poultry/eggs: Bray–Curtis *p* = 0.004) showed abundance associations, while aggregate diet scores did not, supports this interpretation, suggesting that particular dietary components, rather than overall diet quality, may drive abundance shifts. That said, alternative explanations warrant acknowledgment: Sørensen may simply have greater statistical power in sparse communities, and effect sizes were modest (*R*^2^ = 1.9–2.3%). However, the consistency across all three diet scores strengthens confidence in this observation. If the two-stage interpretation holds, dietary optimization may be most effective at establishing beneficial taxa, with complementary approaches (targeted prebiotics or synbiotics) potentially needed to promote their outgrowth.

Caffeine emerged as the sole FDR-significant dietary predictor of cognitive function, likely reflecting its acute pharmacological effects (primarily adenosine receptor antagonism) detectable even cross-sectionally [66], its well-documented neuroprotective properties [67,68,69], and its high variance in this cohort (CV = 79.4%), providing statistical power despite limited MoCA variance. The mediation finding that caffeine’s cognitive effects likely bypass the gut–brain axis, operating through direct neurological mechanisms [70], is mechanistically informative and clinically relevant given that ADT is associated with cognitive impairment [71,72].

The null associations between diet-pattern scores (MIND, MEDAS, and HEI) and MoCA (all *p* > 0.6) likely reflect methodological constraints, including the cross-sectional observational nature of the current analysis, rather than absence of true effects. The restricted MoCA range (11–21 vs. possible 0–30), universal cognitive impairment, and cross-sectional design severely limit power to detect cumulative dietary pattern effects typically observed in longitudinal studies [46,73]. The fact that modifiable factors nonetheless predicted cognition (strength training in the parent study, *p* = 0.007; caffeine here, *p* = 0.0009) suggests that acute or proximal effects remain detectable where cumulative dietary pattern effects are not. Although readily diet-related modifiable pathways to improve cognition beyond caffeine were not revealed in the analysis, the results of the current study are significant for generating hypotheses for future exercise and/or diet intervention studies.

The identification of 129 genera with significant diet associations or diet x host factor interactions (*q* < 0.10) demonstrates that diet profoundly shapes taxonomic composition. Extensive effect modification by exercise (42–55 genera), *APOE* genotype (40–41 genera), and testosterone (34–51 genera) underscores that identical dietary patterns produce different microbiome responses depending on host context. The consistency of certain genera, including *Phascolarctobacterium*, *Butyricimonas*, and *Sutterella*, across all nine analysis categories identifies these taxa as particularly diet-responsive and potential targets for mechanistic studies or intervention monitoring.

Several limitations warrant consideration. The cross-sectional design precludes causal inference; longitudinal studies with dietary interventions are needed to establish whether dietary patterns causally modify the microbiome. The sample size (*n* = 79) limits power for detecting smaller effects and complex interactions, and the E2 carrier subgroup (*n* = 7) is too small for reliable *APOE* e2-specific inference. Self-reported dietary data carry inherent recall bias and social-desirability effects.

Future research should prioritize longitudinal validation to test whether cross-sectional associations predict changes over time, multi-omics integration (metagenomics and metabolomics) to strengthen mechanistic interpretation, randomized dietary intervention trials pairing Mediterranean diet with exercise, and expanded population studies to test replication across cancer types and demographic groups.

Overall, this analysis yields three translational insights. First, the MEDAS × exercise interaction demonstrates that dietary effects on the gut microbiome depend on physical activity context, supporting integrated dietary–exercise counseling over siloed recommendations. Second, the Sørensen–Bray–Curtis dissociation suggests that, at least for this cohort, diet quality affects which taxa colonize rather than their relative abundances, with implications for intervention design. Third, multiple distinct pathways to cognitive support exist: strength training and caffeine both associate with better cognition through different mechanisms, offering complementary targets for survivorship care. Combined with evidence that *APOE* genotype and testosterone status condition microbiome responses to exercise, these findings motivate precision survivorship strategies integrating exercise prescriptions with genotype-, diet-, and hormone-informed monitoring.

## 5. Conclusions

The principal findings of this analysis establish several key patterns in diet–microbiome–cognition relationships among prostate-cancer survivors with ADT exposure. First, the significant MEDAS × exercise interaction on alpha diversity (Shannon *p* = 0.0022) demonstrates that Mediterranean diet adherence shows stronger positive associations with gut microbiome diversity in physically active individuals compared to sedentary controls, supporting the hypothesis that physical activity may prime the gut ecosystem to respond to dietary inputs. Second, the consistent dissociation between beta diversity metrics—where all three diet-quality scores significantly predicted Sørensen distance (all *p* < 0.035) but none predicted Bray–Curtis distance (all *p* > 0.15)—suggests that diet quality shapes community membership rather than abundance dynamics. Third, the extensive taxa-level findings, with 129 unique genera showing significant associations with diet scores or diet × host factor interactions (*q* < 0.10), demonstrate profound diet effects on gut microbial composition that are substantially modified by exercise, *APOE* genotype, and testosterone status. Fourth, among 297 dietary variables tested, only caffeine significantly predicted cognitive function after FDR correction (*p* = 0.0009, *q* = 0.014), with mediation analysis confirming that 97.6% of this effect operates through direct pathways rather than through microbiome modulation. Finally, despite theoretical expectations, the MIND, MEDAS, and HEI diet scores did not predict cognitive function in this cohort, a null finding likely attributable to MoCA range restriction and population homogeneity rather than absence of true diet–cognition relationships. Together, these findings support precision nutrition approaches in cancer survivorship that integrate dietary recommendations with exercise programming and account for individual host characteristics.

## Data Availability

The original contributions presented in this study are included in the article/Supplementary Materials. Further inquiries can be directed to the corresponding authors.

## Supplementary Materials

The following supporting information can be downloaded at https://www.mdpi.com/article/10.3390/microorganisms14010251/s1, Table S1: Details for *APOE* genotyping.

## Abbreviations

AD	Alzheimer’s disease
ADT	androgen-deprivation therapy
*APOE*	*APOLIPOPROTEIN E*
ASV	amplicon sequence variant
ACME	average causal mediation effect
ADE	average direct effect
bp	base pair
CV	coefficient of variance
DHQ	Diet Questionnaire
E2	apolipoprotein E2
E3	apolipoprotein E3
E4	apolipoprotein E4
FDR	False Discovery Rate
HEI-2015	Healthy Eating Index-2015
LASSO	Least Absolute Shrinkage and Selection Operator
MCI	Mild Cognitive Impairment
MaAsLin2	Microbiome Multivariable Associations with Linear Models
MEDAS	Mediterranean Diet Adherence Score
MIND	Mediterranean-Dash Intervention for Neurodegenerative Delay
MoCA	Montreal Cognitive Assessment
PERMANOVA	Permutational Multivariate Analysis of Variance
rRNA	ribosomal RNA
SNP	single-nucleotide polymorphism
