# Supplementary material for: Diet–Microbiome Relationships in Prostate-Cancer Survivors with Prior Androgen Deprivation-Therapy Exposure and Previous Exercise Intervention Enrollment
Source: Microorganisms. 2026 Jan 21;14(1):251. doi: 10.3390/microorganisms14010251 (PMC12843902; doi:10.3390/microorganisms14010251)
Supplement: Supplementary file 1 [file microorganisms-14-00251-s001.zip › microorganisms-4081766-supplementary.pdf]

Suppl. Table S1. Details for *APOE* genotyping

Sample ID	Pate Barcode	Gene Symbol	NCBI SNP Reference	Assay ID	Allele 1 Call	Allele 2 Call	Genotype
APOE 1018 (e3/e3)	CFK0KY7H	APOE	rs429358	C__3084793_20	T	T	TT
APOE 1046 (e4/e4)	CFK0KY7H	APOE	rs429358	C__3084793_20	C	C	CC
APOE 1055 (e4/e3)	CFK0KY7H	APOE	rs429358	C__3084793_20	C	T	CT
APOE IID# 29_OS35 (e2/e3)	CFK0KY7H	APOE	rs429358	C__3084793_20	T	T	TT
APOE 1018 (e3/e3)	CFK0KY71	APOE	rs7412	C__904973_10	C	C	CC
APOE 1046 (e4/e4)	CFK0KY71	APOE	rs7412	C__904973_10	C	C	CC
APOE 1055 (e4/e3)	CFK0KY71	APOE	rs7412	C__904973_10	C	C	CC
APOE IID# 29_OS35 (e2/e3)	CFK0KY71	APOE	rs7412	C__904973_10	C	T	CT
